# Embryo ploidy outcomes of PGT-M+PGT-A compared with PGT-A alone: a multicenter real-world cohort study

**DOI:** 10.3389/fcell.2026.1808395

**Published:** 2026-09-08

**Authors:** Wenjie Huang, Wenchang Lian, Hang Shi, Xiaolan Huang, Peng Zhang, Bao-qiong Liao, Xiaoping Ren, Huihui Xu, Jiaoyan Liu, Kai Wang, Huawei Wang

**Affiliations:** 1 Reproductive Genetics/Obstetrics Department, The First Affiliated Hospital of Kunming Medical University, Kunming, Yunnan, China; 2 Department of Reproductive Medicine, Guangzhou Women and Children’s Medical Center Liuzhou Hospital, Liuzhou, Guangxi, China; 3 Department of Medical Genetics, Yikon Genomics Company Ltd., Suzhou, Jiangsu Province, China; 4 Fujian Maternity and Child Health Hospital, Fuzhou, China; 5 Department of Reproductive Medicine, The Second Affiliated Hospital of Fujian Medical University, Fuzhou, China; 6 Medical Genetics and Prenatal Diagnostic Center, The People’s Hospital of Guangxi Zhuang Autonomous Region, Nanning, China; 7 Ganzhou Maternal and Child Health Hospital, Ganzhou, Jiangxi, China

**Keywords:** aneuploidy, embryo ploidy, PGT-A, PGT-M, preimplantation genetic testing

## Abstract

**Background:**

Given the increasing adoption of concurrent PGT-M + PGT-A from a single biopsy, understanding the baseline embryo ploidy profiles of patients undergoing combined testing compared to standard PGT-A populations is essential, particularly given concerns about embryo attrition. We aimed to compare embryo ploidy outcomes between PGT-M + PGT-A and PGT-A alone across maternal age strata, using PGT-SR cycles as a high-risk reference group.

**Methods:**

This retrospective multicenter real-world cohort included 15,956 PGT cycles performed between January 2021 and August 2024 and analyzed in a single genetics laboratory serving multiple reproductive centers in China (7,635 PGT-A–only; 5,874 PGT-M + PGT-A; 2,447 PGT-SR). Blastocyst trophectoderm biopsies were assessed by next-generation sequencing and classified as euploid, aneuploid, or mosaic. Primary outcomes were cycle-level counts and proportions of each ploidy category. We used Poisson regression for count outcomes and offset-Poisson regression for proportion outcomes (offset = log total embryos tested per cycle), with models prespecified to adjust for female age and to apply cluster-robust standard errors by center. Analyses were repeated across maternal age strata.

**Results:**

Patients undergoing PGT-A alone were older than those undergoing PGT-M + PGT-A (median female age 37 vs. 32 years). After adjustment for female age, euploid proportions were similar between PGT-M + PGT-A and PGT-A alone (RR 0.98, 95% CI 0.95–1.01). Mosaic proportions were slightly higher in PGT-M + PGT-A cycles (RR 1.06, 95% CI 1.002–1.13), whereas aneuploid proportions were slightly lower (RR 0.95, 95% CI 0.90–0.999). Across maternal age strata, the probability of obtaining at least one euploid embryo per cycle was slightly lower among women <35 years (RR 0.96), but did not differ significantly from PGT-A alone in the 35–40 years (RR 1.01) and >40 years (RR 0.87) strata. In contrast, PGT-SR cycles consistently showed poorer euploid outcomes and higher aneuploidy rates, and lower mosaic proportions compared with PGT-A alone.

**Conclusion:**

After adjusting for maternal age, patients undergoing PGT-M + PGT-A exhibited comparable euploid proportions and probabilities of obtaining at least one euploid embryo per cycle to those undergoing PGT-A alone. Because this study assessed embryo ploidy rather than post-transfer outcomes, evaluating the definitive clinical utility of the combined strategy awaits future prospective studies incorporating live birth and miscarriage data.

## Introduction

In the past few decades, the introduction of key technologies—such as blastocyst culture, vitrification, preimplantation genetic testing (PGT), and cycle segmentation (Farquhar and Marjoribanks, 2018) —has enabled IVF treatment to be comprehensively planned and evaluated throughout the entire cycle. Clinical decision-making and outcome evaluation have shifted from focusing solely on “achieving pregnancy” to extending toward the “probability of a healthy live birth within a full treatment cycle.” In this context, patient consultation has gradually become an indispensable core element of assisted reproductive technology (ART). The goal has evolved beyond simply explaining individual techniques to assisting patients in making the most appropriate decisions based on a thorough evaluation of their individual indications and potential benefits. This is especially important when multiple genetic testing indications coexist, as balancing the potential benefits of additional tests with the uncertainty they may introduce has become a key issue in clinical counseling.

PGT is a genetic analysis performed on embryos during *in vitro* fertilization to detect genetic abnormalities and avoid transferring embryos with chromosomal or genetic disorders, thereby reducing adverse pregnancy outcomes. PGT includes preimplantation genetic testing for aneuploidies (PGT-A), monogenic disorders (PGT-M), and structural rearrangements (PGT-SR). PGT-A screens for chromosomal abnormalities to optimize embryo selection, improve pregnancy outcomes, and reduce miscarriage risks. PGT-M prevents the transmission of monogenic diseases, while PGT-SR identifies structural chromosomal abnormalities, minimizing the risk of miscarriage and other pregnancy complications (Alteri et al., 2023). Currently, the clinical application of PGT-M and PGT-SR is typically based on well-defined genetic backgrounds, with clear target populations (2023, Boynukalin et al., 2021; Yan et al., 2023). In contrast, existing research and expert consensus suggest that the clinical application of PGT-A has primarily been proposed and evaluated for specific patient subgroups, such as advanced maternal age (Eliner et al., 2025; Harris et al., 2025), recurrent pregnancy loss (Akin-Olugbade et al., 2025), and repeated implantation failure (Kato et al., 2023; Practice Committees of the American Society for Reproductive and the Society for Assisted Reproductive, 2024). Several studies have explored the clinical effects of PGT-A in other groups, such as its failure to improve outcomes in egg or embryo donation (Dennis et al., 2025; Gingold et al., 2025), and its lack of effect—or even potential adverse impacts—in women under 35 years of age (Harris et al., 2025; Ma et al., 2023). Multicenter prospective randomized studies have shown that PGT-A does not provide benefits for RIF patients under 38 years old (Guan et al., 2025), nor does it improve live birth rates in couples with severe male factor infertility (Lin et al., 2025). Thus, the widespread application of PGT-A is not sufficiently supported by clinical evidence, especially in non-specific patient populations, where its routine use is strongly discouraged (Lensen et al., 2025). However, real-world data indicate that the clinical use of PGT-A has extended beyond the specific populations defined by existing consensus. National data from the United States reveals a steady increase in the proportion of IVF cycles involving PGT in recent years, with approximately 44% of IVF cycles in 2019 incorporating PGT. However, this upward trend does not fully correspond to established medical indications (Eaton, 2022; Gleicher et al., 2025). In China, although expert consensus recommends combining PGT-M with PGT-A as a preferable strategy (Yan et al., 2023), large-scale, real-world data on its baseline ploidy outcomes remain limited.

With advancements in sequencing technology, the simultaneous application of PGT-M + PGT-A on the same embryo biopsy sample has become technically feasible. It is noteworthy that some patients undergoing PGT-M do not have infertility diagnoses and benefit from a younger age profile (Chada et al., 2022; Yan et al., 2023). While combined testing may provide insights into post-embryo transfer pregnancy outcomes, it could also reduce the number of embryos available for transfer and potentially lead to the misidentification of embryos due to mosaicism or false positives (Practice Committees of the American Society for Reproductive and the Society for Assisted Reproductive, 2024). This creates a clear rationale gap regarding whether concurrent PGT-A unnecessarily excludes potentially viable embryos. Recently, Martel et al. reported in a US cohort that age-stratified aneuploidy rates did not significantly differ between PGT-M + PGT-A and PGT-A alone cycles, demonstrating that young patients utilizing the combined strategy were significantly less likely to obtain a usable embryo (Martel et al., 2024). A critical evaluation is therefore required to determine whether these ploidy distributions and attrition trends hold true in a large Chinese multicenter setting.

The primary objective of this study was to compare embryo ploidy outcomes between PGT-M + PGT-A and PGT-A alone across different age groups and genetic inheritance patterns, using PGT-SR as a high-risk reference cohort. We hypothesized that PGT-M patients, who typically lack an infertility diagnosis, would exhibit higher embryonic euploid proportions than age-matched patients undergoing PGT-A alone. By profiling these baseline ploidy distributions, we seek to provide data-driven insights into expected embryo attrition rates, thereby supporting individualized patient counseling for complex PGT indications.

## Materials and methods

### Study population and design

Approval for this study was obtained from the Institutional Review Board of Kunming Medical University First Affiliated Hospital (Ethical Approval 2025L7). The study population included patients who underwent ovarian stimulation and trophectoderm biopsy for preimplantation genetic testing (PGT) at a single genetics laboratory across multiple reproductive centers in China between 1 January 2021, and 31 August 2024. Data received from the laboratory were de-identified to ensure patient confidentiality. The analysis focused on three main PGT strategies: PGT for aneuploidy (PGT-A), combined PGT for monogenic disorders and aneuploidy (PGT-M + PGT-A), and PGT for structural rearrangements (PGT-SR).

Cycles were also stratified according to female age (<35, 35–37, 38–40, 41–42, >42 years). For PGT-SR, to ensure mutually exclusive classification, cycles were stratified into three specific categories based on the primary karyotypic indication: 1) balanced structural rearrangements (Balanced SR; including reciprocal translocations, Robertsonian translocations, and inversions); 2) complex or unbalanced structural rearrangements (Cx/Unbal-SR; including complex translocations, ring chromosomes, and multi-site structural anomalies); and 3) sex chromosome aneuploidies (Sex-Aneu; e.g., 47,XXX; 47,XXY; 47,XYY). Cycles with unknown or unclassified indications were excluded from subtype analyses. For the PGT-M cycles, stratification followed the inheritance modes of the diseases: autosomal recessive (AR), autosomal dominant (AD), X-linked recessive (XLR), and X-linked dominant (XLD).

Inclusion criteria were patients undergoing PGT-A alone, PGT-M + PGT-A, or PGT-SR with available data for embryo ploidy. Exclusion criteria included cycles utilizing oocyte donation, cycles undergoing PGT-M without concurrent PGT-A analysis (as our specific objective was to evaluate the combined PGT-M + PGT-A strategy directly against the standard PGT-A alone baseline), and cycles in which the genetic inheritance mode of monogenic diseases was unclear (non-AR, AD, XLR, or XLD). Cycles with concurrent indications for PGT-M and structural chromosomal rearrangements were classified as PGT-SR and were not included in the PGT-M + PGT-A group.

### Embryo biopsy and NGS analysis

Embryos reaching the blastocyst stage were biopsied for genetic analysis, including PGT-A, PGT-M, and PGT-SR. For PGT-A, trophectoderm biopsy was performed by stabilizing the embryo with a holding pipette and using a laser system (ZILOS-tk Laser, Hamilton Thorne Inc., Beverly, MA, USA) to perforate the zona pellucida. A biopsy pipette (20-μm internal diameter) was then used to aspirate 3–10 cells from the trophectoderm.

Biopsied cell samples underwent whole-genome amplification (WGA) and library construction using the general-purpose sequencing library kit (XK-008, Yikon Genomics). The amplification utilized a quasi-linear method with primers containing a 27-nucleotide common sequence at the 5′end and a variable 8-nucleotide sequence at the 3′end. Circularized amplicons were formed to prevent allele drop-out (ADO) during PCR. The library was fragmented (200–1,000 bp) and sequenced on the Illumina NextSeq550AR platform, yielding approximately 1.2 million reads.

Copy Number Variation (CNV) Analysis and Mosaicism Calling: After removing duplicate reads, raw reads were aligned to the genome in 1 Mb bins, and counts normalized based on GC content. CNVs were detected using the circular binary segmentation (CBS) algorithm with a minimum resolution of 4 Mb for both full and segmental aneuploidies, and visualized across 24 chromosomes using an R-based program. Embryos were classified into three ploidy categories based on the fraction of aneuploid cells: euploid (<20%), mosaic (20%–80%), and aneuploid (>80%). Although the software identified both segmental and whole-chromosome mosaicisms, they were pooled into a single comprehensive “mosaic” category for cycle-level statistical analyses to evaluate overall embryo attrition.

Amplification and Library Preparation: Biopsied samples underwent amplification and library preparation using one of three distinct methodological categories based on clinical indications: (1) One-step library preparation (e.g., ChromInst, SurePlex), a streamlined approach predominantly used for PGT-A and a subset of PGT-SR; (2) Two-step/WGA-based methods (e.g., ChromSwift™, MDA), using φ29 DNA polymerase for robust whole-genome amplification (WGA) via a quasi-linear approach; and (3) Long-fragment methods (e.g., Chromlong™), which generated DNA fragments (50–200 kb) suitable for large-insert libraries, ensuring high fidelity for complex SNV analysis in PGT-M.

For samples undergoing WGA (Two-step or Long-fragment methods), a 20 ng portion of the WGA product was fragmented and sequenced at 0.04X depth to assess chromosomal aneuploidy. Additionally, 120 ng was used for multiplex amplification of SNP primers targeting pathogenic genes, followed by library construction with the gene sequencing kit (XK-038, Yikon Genomics), achieving a sequencing depth of 100X for accurate SNP detection.

Amplified DNA was analyzed using Illumina NGS technology (BlueGnome, Cambridge, United Kingdom), aligned to a reference genome, and processed using ChromGO software (Yikon version 1.8.3). Embryos were classified as euploid, aneuploid, or mosaic, and those without disease-causing mutations were recommended for clinical transfer.

### Statistical analysis

Study outcomes included the numbers and proportions of aneuploid, euploid, and mosaic embryos per ovarian stimulation cycle, as well as the number of biopsied blastocysts per cycle. Poisson regression was used to evaluate associations between PGT strategy and count outcomes (biopsied blastocysts and numbers of euploid, aneuploid, and mosaic embryos), with results reported as rate ratios (RRs) and 95% confidence intervals (CIs). Proportion outcomes were analyzed using Poisson regression with an offset for the log of the total number of embryos tested per cycle [offset (log (total))], and results were also reported as RRs with 95% CIs. All models were specified a priori to adjust for female age, and cluster-robust standard errors by center were applied. Subgroup analyses by female age strata were performed using the same modeling framework. Outcome variables were complete in the analytic dataset; missing values were confined to covariates (female age, male age, and season) and were handled using multiple imputation by chained equations. Analyses were conducted in R (R Foundation for Statistical Computing, Vienna, Austria).

## Results

A total of 15,956 PGT cycles were included in the analysis, comprising 7,635 cycles undergoing PGT-A alone, 5,874 cycles undergoing combined PGT-M and PGT-A, and 2,447 cycles undergoing PGT-SR.

Significant differences in patient age were observed across the three PGT strategies. Patients undergoing PGT-A alone were substantially older than those undergoing PGT-M + PGT-A or PGT-SR. The median female age was 37 years (interquartile range [IQR], 33–40) in the PGT-A alone group, compared with 32 years (IQR, 29–35) in the PGT-M + PGT-A group and 31 years (IQR, 29–34) in the PGT-SR group (both p < 0.001). A similar pattern was observed for male age, with median ages of 37 years (IQR, 34–41), 33 years (IQR, 30–36), and 32 years (IQR, 30–35), respectively (both p < 0.001).

The age distribution differed markedly among the three groups. Patients aged younger than 35 years accounted for the majority of cycles in the PGT-M + PGT-A and PGT-SR groups, whereas a substantially higher proportion of patients aged 38 years or older was observed in the PGT-A alone group (Table 1).

**TABLE 1:** Demographic characteristics of patients undergoing PGT-A alone, PGT-M + PGT-A, and PGT SR.

Characteristic	PGT-A alone (N = 7,635)	PGT-m+pgt-A (N = 5,874)	p-value	PGT-SR (N = 2,447)	p-value
Female age, median (IQR)	37 (33–40)	32 (29–35)	<0.001	31 (29–34)	<0.001
Age group, No. (%)		​	​	​	​
<35	2,562 (33.56%)	4,334 (73.78%)	​	1905 (77.85%)	​
35–37	1722 (22.55%)	957 (16.29%)	​	330 (13.49%)	​
38–40	1846 (24.18%)	409 (6.96%)	​	162 (6.62%)	​
41–42	938 (12.29%)	133 (2.26%)	​	29 (1.19%)	​
>42	567 (7.43%)	41 (0.7%)	​	21 (0.86%)	​
Male age, median (IQR)	37 (34–41)	33 (30–36)	<0.001	32 (30–35)	<0.001
Season, No. (%)		​	<0.001	​	<0.001
spring	1756 (23%)	1,391 (23.68%)	​	206 (8.42%)	​
Summer	2,650 (34.71%)	2,245 (38.22%)	​	305 (12.46%)	​
Autumn	2,492 (32.64%)	1,539 (26.2%)	​	1,022 (41.77%)	​
Winter	737 (9.65%)	699 (11.9%)	​	914 (37.35%)	​

PGT-A, preimplantation genetic testing for aneuploidy; PGT-M, preimplantation genetic testing for monogenic disorders; PGT-SR, preimplantation genetic testing for structural rearrangements. Median (interquartile range) is reported for age variables. Chi-square test was used for categorical comparisons; Kruskal–Wallis test was used for continuous variables. P-values less than 0.05 were considered statistically significant.

In addition, the distribution of treatment cycles across seasons differed significantly among the three PGT strategies (p < 0.001). The PGT-A alone and PGT-M + PGT-A cycles were more frequently performed in spring and summer, whereas a higher proportion of PGT-SR cycles occurred in autumn and winter (Table 1).

Table 2 summarizes blastocyst biopsy and ploidy outcomes by PGT strategy. Descriptive values represent unadjusted mean estimates, whereas all regression analyses were specified a priori to adjust for female age and used cluster-robust standard errors by center. For count outcomes, despite higher unadjusted mean numbers of biopsied blastocysts and euploid embryos in the PGT-M + PGT-A group, age-adjusted analyses showed that PGT-M + PGT-A cycles had similar biopsied blastocysts (RR 1.02, 95% CI 0.97–1.08), more euploid embryos (RR 1.18, 95% CI 1.10–1.27), and more mosaic embryos (RR 1.24, 95% CI 1.15–1.34) compared with PGT-A alone, while aneuploid embryo counts were significantly lower (RR 0.73, 95% CI 0.68–0.79). PGT-SR cycles had more aneuploid embryos (RR 1.81, 95% CI 1.68–1.95) and fewer euploid embryos (RR 0.76, 95% CI 0.70–0.83). For proportion outcomes, offset-Poisson models showed higher mosaic proportions with PGT-M + PGT-A (RR 1.06, 95% CI 1.002–1.13) but no clear difference in euploid proportions (RR 0.98, 95% CI 0.95–1.01). The association with aneuploid proportion was negative in unadjusted analyses (RR 0.72, 95% CI 0.68–0.76) but became attenuated towards the null after adjustment for female age (adjusted RR 0.95, 95% CI 0.90–0.999), an attenuation consistent with strong age confounding (Supplementary Figure 1). PGT-SR was associated with lower euploid (RR 0.53, 95% CI 0.50–0.56) and higher aneuploid proportions (RR 2.13, 95% CI 2.01–2.26), as well as lower mosaic proportions (RR 0.73, 95% CI 0.67–0.81). These associations were similar after additional adjustment for male age (Supplementary Table 1), treatment season (Supplementary Table 2), or both (Supplementary Table 3).

**TABLE 2:** Association between ploidy and outcomes of PGT-A alone, PGT-M + PGT-A, and PGT SR.

Ploidy	PGT-A alone (N = 7,635)	PGT- M + PGT-A (N = 5,874)	PGT-SR (N = 2,447)
Biopsied blastocysts	3.51 (3.46–3.57)	3.60 (3.53–3.67)	4.13 (4.02–4.25)
	Ref (1.0)	1.02 (0.97–1.08)	1.18 (1.11–1.25)
Aneuploid embryos	1.30 (1.27–1.33)	0.95 (0.92–0.98)	2.34 (2.26–2.42)
	Ref (1.0)	0.73 (0.68–0.79)	1.81 (1.68–1.95)
Mosaic embryos	0.44 (0.42–0.45)	0.54 (0.52–0.56)	0.44 (0.41–0.47)
	Ref (1.0)	1.24 (1.15–1.34)	1.00 (0.92–1.10)
Euploid embryos	1.78 (1.74–1.82)	2.11 (2.05–2.16)	1.35 (1.29–1.41)
	Ref (1.0)	1.18 (1.10–1.27)	0.76 (0.70–0.83)
Aneuploid (%)	41.34 (40.51–42.17)	28.29 (27.44–29.14)	57.38 (56.04–58.72)
	Ref (1.0)	0.95 (0.90–0.999)	2.13 (2.01–2.26)
Mosaic (%)	12.18 (11.68–12.68)	14.97 (14.34–15.61)	10.90 (10.08–11.71)
	Ref (1.0)	1.06 (1.002–1.13)	0.73 (0.67–0.81)
Euploid (%)	46.48 (45.66–47.3)	56.78 (55.82–57.65)	31.72 (30.48–32.97)
	Ref (1.0)	0.98 (0.95–1.01)	0.53 (0.50–0.56)

Data are presented as mean (95% confidence interval) for descriptive summaries.

For count outcomes (number of biopsied blastocysts and numbers of euploid, aneuploid, and mosaic embryos per cycle), Poisson regression models were fitted with a priori adjustment for female age and cluster-robust standard errors by center; results are reported as rate ratios (RRs) with 95% CIs, using PGT-A, alone as the reference.

For proportion outcomes (aneuploid, mosaic, and euploid proportions), embryo-level rates were modeled using Poisson regression with an offset for the log of the total number of embryos tested per cycle [offset (log (total))], with a priori adjustment for female age and cluster-robust standard errors by center; results are reported as RRs (95% CIs), interpreted as the relative difference in the proportion (rate) of embryos with each ploidy category compared with PGT-A, alone.

**FIGURE 1:**
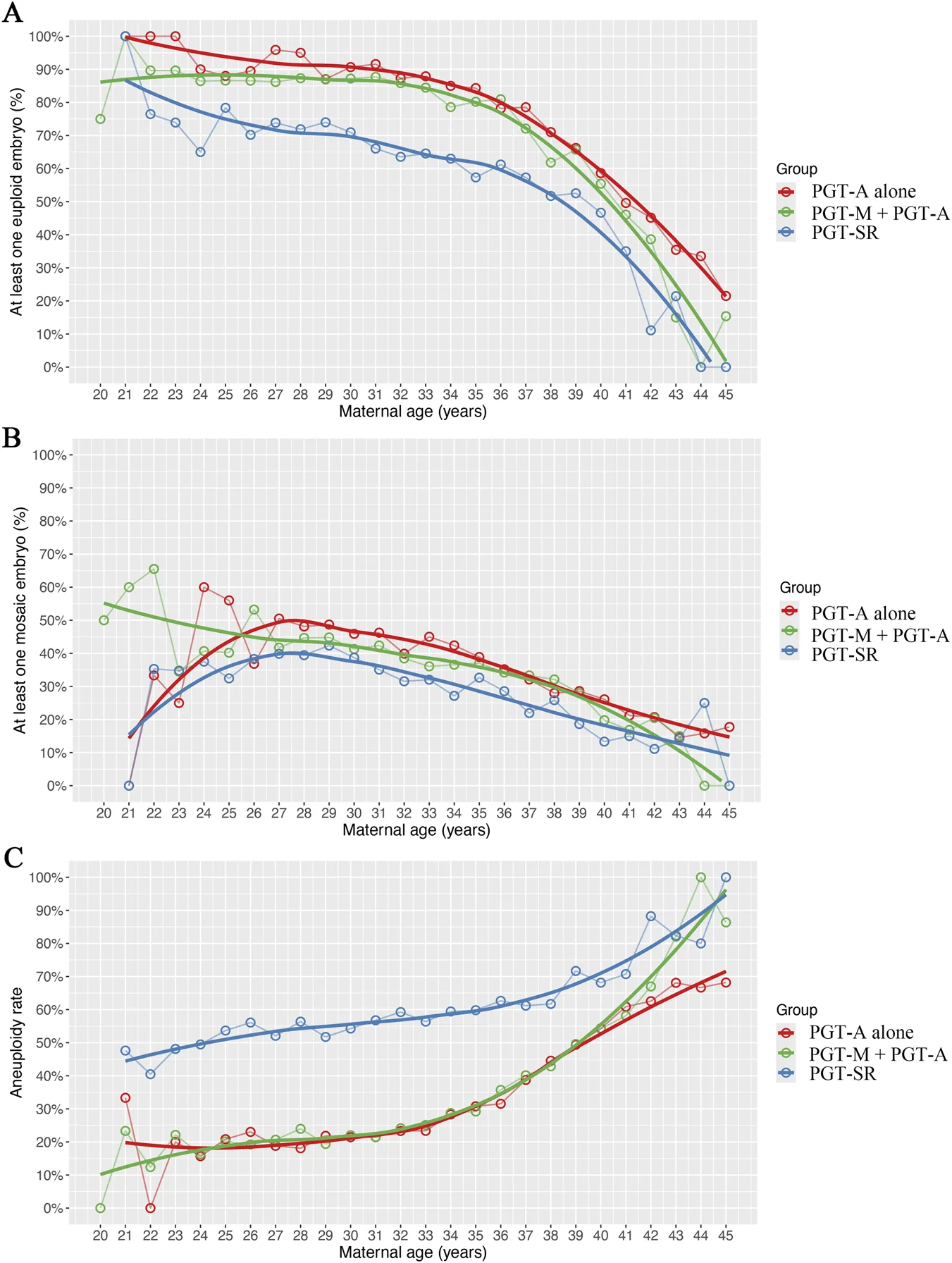
Maternal age–related biopsy outcomes across different PGT strategies. **(A)** Proportion of cycles with at least one euploid embryo according to maternal age. **(B)** Proportion of cycles with at least one mosaic embryo according to maternal age. **(C)** Aneuploidy rate according to maternal age. Solid lines represent smoothed conditional means fitted by LOESS to illustrate overall age-related trends.

Age-stratified descriptive trends in cycle-level ploidy outcomes are shown in Figure 1. Across maternal age groups, the proportion of cycles achieving at least one euploid blastocyst appeared similar between PGT-M + PGT-A and PGT-A alone (Figure 1A), whereas consistently lower proportions were observed in PGT-SR cycles. The proportion of cycles with at least one mosaic blastocyst varied across PGT strategies and maternal age (Figure 1B), with similar proportions in PGT-M + PGT-A cycles compared with PGT-A alone and lower proportions in PGT-SR cycles. Aneuploidy rates increased with advancing maternal age across all strategies and were highest in PGT-SR cycles (Figure 1C). In formal Poisson regression analyses of cycle-level binary outcomes (Supplementary Table 4), PGT-M + PGT-A was associated with a slightly lower likelihood of achieving at least one euploid blastocyst among women aged <35 years (RR 0.96, 95% CI 0.95–0.98), but did not differ significantly from PGT-A alone in the 35–40 years (RR 1.01, 95% CI 0.96–1.06) and >40 years (RR 0.87, 95% CI 0.69–1.09) age strata. In contrast, PGT-SR was consistently associated with a lower likelihood of achieving at least one euploid blastocyst across all age groups, with relative risks ranging from 0.53 to 0.78. With respect to mosaic outcomes, cycles undergoing PGT-M combined with PGT-A were associated with a slightly lower likelihood of achieving at least one mosaic blastocyst among women aged <35 years (RR 0.93, 95% CI 0.87–0.99), but showed no significant difference compared with PGT-A alone in the 35–40 years (RR 1.05, 95% CI 0.96–1.15) and >40 years (RR 0.82, 95% CI 0.54–1.24) age strata. PGT-SR was consistently associated with a lower likelihood of achieving at least one mosaic blastocyst compared with PGT-A alone, with the reduction being statistically significant among women aged <35 years (RR 0.80, 95% CI 0.74–0.86) and 35–40 years (RR 0.82, 95% CI 0.71–0.95).

Table 3 presents age-stratified embryo ploidy proportions by PGT strategy. Across all strategies, euploid proportions decreased and aneuploid proportions increased with advancing female age. Within each age stratum, PGT-SR was consistently associated with a markedly lower euploid proportion and a higher aneuploid proportion compared with PGT-A alone. For PGT-M + PGT-A, euploid proportions were broadly comparable to PGT-A alone across the younger and mid-age strata (<35, 35–37, and 38–40 years; RRs close to 1.0), and mosaic proportions were also comparable. Notably, despite the lower crude aneuploid proportion observed in unstratified descriptive summaries, age-stratified analyses showed that PGT-M + PGT-A was associated with comparable aneuploid proportions relative to PGT-A alone in the <35, 35–37, and 38–40 years strata, highlighting substantial confounding by maternal age due to the markedly different age distributions between groups. Estimates in the oldest age strata were less precise because of smaller sample sizes. Supplementary Table 5 presents the corresponding age-stratified count outcomes, which similarly showed declining euploid embryo numbers with increasing female age across strategies.

**TABLE 3:** Association between PGT strategy and embryo ploidy proportions within female age subgroups.

Ploidy	PGT-A alone	PGT-m + PGT-A	PGT-SR
<35	​	​	​
Aneuploid (%)	23.13 (22.07–24.19)	23.37 (22.48–24.25)	55.26 (53.76–56.76)
	Ref (1.0)	0.99 (0.93–1.05)	2.40 (2.26–2.56)
Mosaic (%)	15.20 (14.31–16.08)	15.73 (14.99–16.48)	11.22 (10.30–12.13)
	Ref (1.0)	1.06 (0.99–1.14)	0.74 (0.67–0.82)
Euploid (%)	61.68 (60.45–62.91)	60.90 (59.88–61.91)	33.52 (32.10–34.93)
	Ref (1.0)	0.99 (0.97–1.01)	0.54 (0.51–0.57)
35–37	​	​	​
Aneuploid (%)	34.04 (32.46–35.61)	33.93 (31.72–36.14)	61.45 (57.80–65.10)
	Ref (1.0)	1.02 (0.95–1.09)	1.82 (1.68–1.96)
Mosaic (%)	13.24 (12.13–14.36)	13.90 (12.37–15.44)	10.40 (8.11–12.70)
	Ref (1.0)	1.10 (0.99–1.23)	0.76 (0.63–0.90)
Euploid (%)	52.72 (51.07–54.37)	52.16 (49.86–54.47)	28.15 (24.85–31.44)
	Ref (1.0)	0.97 (0.92–1.01)	0.55 (0.49–0.61)
38–40	​	​	​
Aneuploid (%)	51.47 (49.80–53.14)	50.08 (46.34–53.81)	66.58 (61.24–71.91)
	Ref (1.0)	0.97 (0.88–1.06)	1.36 (1.27–1.46)
Mosaic (%)	10.29 (9.34–11.25)	12.39 (10.07–14.72)	9.14 (5.71–12.58)
	Ref (1.0)	1.19 (1.01–1.40)	0.84 (0.59–1.20)
Euploid (%)	38.24 (36.63–39.84)	37.53 (33.95–41.11)	24.28 (19.55–29.01)
	Ref (1.0)	0.99 (0.89–1.10)	0.60 (0.52–0.69)
41–42	​	​	​
Aneuploid (%)	64.77 (62.45–67.10)	61.39 (54.57–68.21)	81.26 (70.77–91.76)
	Ref (1.0)	0.99 (0.88–1.11)	1.23 (1.07–1.42)
Mosaic (%)	8.65 (7.32–9.97)	9.79 (5.67–13.92)	5.00 (0.0–10.01)
	Ref (1.0)	0.98 (0.67–1.44)	0.77 (0.35–1.69)
Euploid (%)	26.58 (24.42–28.73)	28.81 (22.46–35.17)	13.74 (4.54–22.93)
	Ref (1.0)	1.02 (0.82–1.27)	0.59 (0.36–0.96)
>42	​	​	​
Aneuploid (%)	74.09 (71.12–77.05)	92.28 (86.10–98.46)	81.75 (66.14–97.35)
	Ref (1.0)	1.28 (1.09–1.50)	1.23 (0.99–1.52)
Mosaic (%)	7.25 (5.58–8.92)	2.03 (0.0–4.30)	11.11 (0.0–24.13)
	Ref (1.0)	0.51 (0.18–1.43)	0.93 (0.29–3.00)
Euploid (%)	18.66 (16.10–21.23)	5.69 (0.77–10.61)	7.14 (0.0–15.14)
	Ref (1.0)	0.37 (0.15–0.95)	0.39 (0.12–1.27)

Within each female age subgroup, descriptive values are presented as mean proportions (95% confidence intervals). Regression estimates in the >42 age group were imprecise because of limited sample size, and should therefore be interpreted with caution.

Formal comparisons of aneuploid, mosaic, and euploid proportions across PGT, strategies were conducted using Poisson regression with an offset for the log of the total number of embryos tested per cycle [offset (log (total))] and cluster-robust standard errors by center. Results are reported as RRs (95% CIs) with PGT-A, alone as the reference and may be interpreted as relative differences in embryo-level rates (proportions) within each age stratum.

Table 4 presents the probability of achieving at least one euploid embryo per cycle among PGT-A alone and inheritance subtypes within the PGT-M + PGT-A group, stratified by female age (<35, 35–40, and >40 years) and the number of embryos available for biopsy. Across all groups, the probability of obtaining at least one euploid embryo decreased with increasing female age and increased with a higher number of embryos for biopsy. Within most age-by-embryo strata, the probability estimates for PGT-M + PGT-A inheritance subtypes were generally comparable to those for PGT-A alone, with statistically significant differences observed only in selected strata after Bonferroni correction (Table 4). Supplementary Table 6 presents analogous subtype analyses for PGT-SR. Compared with PGT-A alone, PGT-SR—particularly balanced structural rearrangements—was consistently associated with a lower probability of achieving at least one euploid embryo across age strata and embryo-number strata. In contrast, estimates for less common PGT-SR subtypes (such as complex/unbalanced rearrangements or sex chromosome aneuploidies) were limited by small sample sizes in several strata, though they also demonstrated lower euploid probabilities where statistical estimation was possible.

**TABLE 4:** Probability of achieving at least one euploid embryo per cycle among PGT-A alone and PGT-M + PGT-A subtypes, stratified by female age and number of embryos for biopsy.

Female age/EMFB	PGT-A alone % (SE)	PGT-M + PGT-A % (SE)				p-value comparing to PGT-alone			
Age	n = 7,635	AD = 1921	AR = 2,905	XLD = 84	XLR = 605	AD	AR	XLD	XLR
<35	N = 2,562	N = 1,464	N = 2077	N = 71	N = 442	​	​	​	​
Combined	88.72 (0.62)	84.63 (0.94)	86.66 (0.75)	87.32 (3.95)	85.29 (1.68)	<0.001	0.022	0.730	0.051
1–3 EMFB	77.16 (1.23)	74.85 (1.49)	76.88 (1.25)	76.32 (6.9)	75 (2.75)	0.180	0.845	0.900	0.486
4–6 EMFB	97.46 (0.52)	97.55 (0.77)	97.7 (0.61)	100 (0)	97.54 (1.4)	0.925	0.772	<0.001	0.959
>7 EMFB	99.8 (0.2)	99.04 (0.67)	99.7 (0.3)	100 (0)	100 (0)	0.423	0.783	0.313	0.313
35–40	N = 3,568	N = 421	N = 726	N = 11	N = 134	​	​	​	​
Combined	72.59 (0.75)	72.68 (2.17)	73.55 (1.64)	81.82 (11.63)	73.88 (3.79)	0.967	0.650	0.359	0.739
1–3 EMFB	60.25 (1.04)	63.67 (2.73)	64.01 (2.17)	75 (15.31)	64.13 (5)	0.216	0.134	0.283	0.392
4–6 EMFB	89.79 (0.96)	97.44 (1.79)	91.48 (2.1)	3/3	93.94 (4.15)	<0.001	0.496	-	0.293
>7 EMFB	99.73 (0.27)	100 (0)	98.36 (1.63)	-	100 (0)	0.323	0.398	-	0.323
>40	N = 1,505	N = 36	N = 102	N = 2	N = 39	​	​	​	​
Combined	41.79 (1.27)	38.89 (8.12)	38.24 (4.81)	0/2	31.03 (8.59)	0.698	0.483	-	0.293
1–3 EMFB	31.44 (1.36)	34.38 (8.4)	30.95 (5.04)	0/2	21.74 (8.6)	0.685	0.937	-	0.320
4–6 EMFB	72.79 (2.65)	2/3	72.22 (10.56)	-	66.67 (19.25)	-	0.941	-	0.745
>7 EMFB	95.08 (2.77)	1/1	-	-	0/1	-	-	-	-

AD, autosomal dominant; AR, autosomal recessive; XLD = X-linked dominant; XLR = X-linked recessive; EMFB, embryos for biopsy; SE, standard error. Values are presented as the percentage of cycles with at least one euploid embryo, with SEs, shown in parentheses. Results are stratified by female age group (<35, 35–40, and >40 years) and number of embryos biopsied per cycle (1–3, 4–6, and >7). Comparisons between each PGT-M + PGT-A, inheritance subtype (AD, AR, XLD, and XLR) and PGT-A, alone were performed within each age and EMFB, stratum. Relative risks were estimated using Poisson regression models with robust standard errors clustered by center. P values shown are unadjusted. For the four subtype comparisons within each age-by-EMFB, stratum, a Bonferroni-adjusted significance threshold of 0.0125 was used to determine statistical significance (two-sided). A total of 359 PGT-M + PGT-A, cycles with missing or unclassified inheritance subtype were excluded from the subtype-stratified analyses and are not shown in the AD/AR/XLD/XLR, columns. For strata with zero events or fewer than five cycles (N < 5), statistical estimation is unreliable. Consequently, data in these cells are presented as raw counts (n/N) without percentages or standard errors, and corresponding p-values are omitted and marked with dashes.

## Discussion

With advances in sequencing technology, combined PGT-M and PGT-A from a single biopsy has become increasingly routine, highlighting the need to understand the ploidy profiles of patients undergoing combined testing compared to standard PGT-A indications. As expected, PGT-SR cycles showed consistently poorer ploidy outcomes, including lower euploid proportions and higher aneuploidy. In contrast, after adjustment for maternal age, euploid proportions and the probability of achieving at least one euploid embryo per cycle were comparable between PGT-M + PGT-A and PGT-A alone, with similarly comparable mosaic proportions between the two strategies. Notably, PGT-SR cycles were consistently associated with significantly lower mosaic proportions; therefore, recognizing this baseline parity is crucial for individualized patient counseling regarding potential embryo attrition and diagnostic uncertainty.

Our findings align with previous studies showing that the likelihood of obtaining euploid or transferable embryos in PGT-SR cycles remains low (Boynukalin et al., 2021; Mateu-Brull et al., 2019; Tan et al., 2025), with nearly half of cycles yielding no usable embryos (Jia et al., 2024). Even in balanced translocation carriers, the proportion of euploid embryos is significantly lower than in standard PGT-A populations, with many showing interchromosomal effects (Nakano et al., 2022).

Our ploidy-centric findings provide relevant baseline data for counseling patients contemplating PGT-M + PGT-A, echoing recent recommendations that emphasize individualized discussions over automatic inclusion (Gleicher et al., 2025; Practice Committees of the American Society for Reproductive and the Society for Assisted Reproductive, 2024). Moreover, Li et al. observed that 26.5% of embryos free of the pathogenic variant were still aneuploid (Li et al., 2018), and Toft et al. similarly estimated that approximately one-third of embryos in PGT-M cycles exhibit aneuploidy (Toft et al., 2020), underscoring that adding PGT-A can exclude a substantial proportion of embryos even after monogenic screening. Despite their significantly younger demographic, patients in the PGT-M + PGT-A cohort yielded a comparable, rather than vastly superior, overall number of biopsied blastocysts (adjusted RR 1.02), and even slightly fewer blastocysts within matched younger age strata. This likely reflects clinical strategies tailored to younger patients—such as mild stimulation to prevent OHSS or cost-driven partial biopsies—rather than diminished biological reserve. Regarding embryonic aneuploidy, our unadjusted rates showed a lower overall aneuploidy proportion in the PGT-M + PGT-A group (28.29% vs. 41.34%). However, given the profound, non-linear increase in embryonic aneuploidy with advancing maternal age (Franasiak et al., 2014), controlling for this demographic imbalance is critical. After age adjustment, the trend was attenuated towards the null, revealing comparable or slightly lower baseline aneuploid proportions in our combined cohort relative to PGT-A alone. This finding aligns with a recent large-scale US-based study by Martel et al., which also reported equivalent age-stratified aneuploidy rates between PGT-M + PGT-A and PGT-A alone groups (Martel et al., 2024). The consistency between these two large, independent cohorts across different genetic backgrounds and national practices strongly suggests that despite their theoretically more favorable clinical characteristics (e.g., a general lack of primary infertility), PGT-M patients do not inherently possess a superior embryonic ploidy profile. Instead, once maternal age is accounted for, their aneuploid and euploid proportions are fundamentally comparable to those of the standard PGT-A population, with euploid probabilities even appearing lower in the most advanced age strata.

We observed comparable mosaic proportions between the PGT-M + PGT-A and PGT-A alone cohorts across all age strata, whereas the PGT-SR cohort exhibited the lowest mosaicism rate (adjusted RR 0.73). This aligns with the biology of structural chromosomal rearrangements: meiotic segregation predominantly produces gametes with uniform segmental aneuploidies rather than post-zygotic mitotic errors (Viotti, 2020). Consequently, abnormal embryos in the PGT-SR group are overwhelmingly classified as fully aneuploid, which proportionally dilutes the mosaic rate in this cohort. We also found that the probability of obtaining at least one euploid embryo was not significantly different across PGT-M inheritance patterns, with the likelihood primarily increasing as the number of biopsied embryos increased. This is consistent with previous findings that the number of oocytes and biopsied embryos, rather than the genetic inheritance pattern, plays a larger role in cycle outcomes (Ben-Nagi et al., 2019; Hu et al., 2015). Further analysis showed that when “available embryos” were defined as both euploid and free of pathogenic genes, differences between inheritance patterns became more pronounced, especially in autosomal dominant and X-linked diseases, where a higher proportion of embryos are affected, leading to a reduced number of available embryos (Martel et al., 2024).

It is important to note that being classified as aneuploid or mosaic does not imply complete loss of reproductive potential. Studies show that some chromosomally aneuploid embryos, especially those with segmental abnormalities, may still achieve reasonable live birth rates under specific conditions (Madjunkov et al., 2026). Multicenter studies confirm that while pregnancy and live birth rates for mosaic embryos are lower than for euploid embryos, a certain proportion still achieve clinical pregnancy and healthy live birth (Jin et al., 2026). Moreover, recent case reports show that embryos classified as aneuploid by PGT-A can, in some cases, develop into healthy offspring with normal chromosomal profiles, suggesting discrepancies between trophectoderm biopsy and the true chromosomal makeup (Tise et al., 2025). These findings challenge the static interpretation of embryo ploidy. Orvieto et al. suggested that aneuploid or mosaic status may reflect a dynamic phase of development, where some embryos can self-correct chromosomal abnormalities (Orvieto et al., 2020). Recent views argue that PGT-A should be used as a risk stratification tool rather than a definitive outcome determinant, as over-reliance on binary classifications may oversimplify the interpretation of developmental potential (Cooper and Viotti, 2024). These insights call for a reconsideration of the necessity and applicability of routinely adding PGT-A in all PGT-M cycles.

A common argument for PGT-M + PGT-A is its potential to improve post-transfer outcomes, especially in younger women, with higher implantation and lower miscarriage rates (Hou et al., 2019; Treff and Zimmerman, 2017). However, these studies included only cycles with embryos that completed transfer, and the PGT-M cohort typically consisted of younger women, many without clear infertility diagnoses, with the primary goal of avoiding genetic diseases (Chada et al., 2022). In this context, the combined strategy’s potential benefits are more apparent. Theoretically, patients undergoing PGT-M + PGT-A, given their younger age structure and clear genetic screening goals, would be expected to exhibit lower baseline aneuploidy rates compared to the standard PGT-A population. Additionally, previous studies show that PGT-M has high acceptance in high-risk genetic populations, with patients valuing its information quality and accuracy, further boosting confidence after a live birth outcome (Lee et al., 2020). In clinical practice, patients often prioritize post-transfer outcomes and may overlook the risk of “no available embryos” when opting for PGT-M + PGT-A. However, real-world data show that many PGT-M + PGT-A cycles fail to proceed to transfer due to a lack of viable embryos (Stocker et al., 2022; Willson et al., 2024). Moreover, current evidence does not support a direct link between most monogenic diseases and increased aneuploidy risk (Fan et al., 2026; Lee et al., 2026). Therefore, higher screening intensity does not necessarily lead to better reproductive outcomes.

The strength of this study lies in its use of large-scale real-world data, comparing the embryo ploidy outcomes of PGT-A, PGT-M + PGT-A, and PGT-SR strategies under standardized laboratory conditions, which reduces the impact of platform and algorithm differences. We adjusted for female age, employed cluster-robust standard errors, and conducted multiple stratified analyses, ensuring consistent and interpretable results across age groups and embryo strata. The primary conclusions remained stable after further adjustments for male age and treatment season, suggesting robustness.

However, this study has limitations. The retrospective observational design may still be subject to residual confounding. Because data were extracted from a centralized genetics laboratory, we lacked comprehensive demographic and upstream clinical data; consequently, we were unable to adjust for specific predictors of blastocyst ploidy—such as detailed infertility diagnoses, ovarian reserve markers, stimulation protocols, and sperm parameters—which also limits further analysis of population differences. Additionally, the study focused on embryo ploidy outcomes, lacking downstream clinical data, which prevents direct assessment of the combined strategy’s effect on cumulative reproductive success.

Future research should use prospective designs that integrate embryo ploidy and post-transfer outcomes (cumulative pregnancy, live birth, and perinatal outcomes) to more comprehensively assess the true benefits and costs of routinely adding PGT-A in PGT-M cycles. It will also be essential to factor in female age, embryo quantity, and genetic inheritance patterns to identify subgroups that may benefit from combined testing. This would avoid equating high screening intensity with superior reproductive outcomes.

In current clinical practice, PGT-M + PGT-A has been widely used in embryo selection and transfer decisions. Our study, based on large-scale real-world data under standardized testing conditions, provides empirical evidence for reconsidering this strategy’s applicability in different populations.

## Conclusion

In conclusion, this large multicenter real-world study demonstrates that after accounting for maternal age, patients undergoing combined PGT-M + PGT-A yield euploid proportions, aneuploid proportions, mosaic proportions, and probabilities of obtaining at least one euploid embryo per cycle that are broadly comparable to those observed in patients undergoing PGT-A alone. Conversely, PGT-SR cycles are characterized by substantially higher aneuploidy and correspondingly lower euploidy and mosaicism. Because our study assessed embryo ploidy rather than post-transfer outcomes such as live birth or miscarriage, these findings solely reflect laboratory cycle parameters and cannot dictate clinical practice. Evaluating the true clinical utility of routinely adding PGT-A to PGT-M cycles will require further prospective studies that incorporate these downstream reproductive endpoints.

## Data Availability

The original contributions presented in the study are included in the article/Supplementary Material, further inquiries can be directed to the corresponding authors.
