# Supplementary material for: Embryo ploidy outcomes of PGT-M+PGT-A compared with PGT-A alone: a multicenter real-world cohort study
Source: Front Cell Dev Biol. 2026 Sep 8;14:1808395. doi: 10.3389/fcell.2026.1808395 (PMC13593825; doi:10.3389/fcell.2026.1808395)
Supplement: Supplementary file 1 [file Table1.DOCX]

**Supplementary table 1.** Association between ploidy and outcomes of PGT-A, PGT-M+PGT-A, and PGT SR with additional adjustment for male age.

| Ploidy | PGT-A (N=7635) | PGT-M+PGT-A (N=5874) | PGT-SR (N=2447) |
| --- | --- | --- | --- |
| Biopsied blastocysts | Ref (1.0) | 0.869 (0.824 - 0.917) | 0.976 (0.919 - 1.037) |
| Aneuploid embryos | Ref (1.0) | 0.822 (0.761 - 0.887) | 2.054 (1.892 - 2.230) |
| Mosaic embryos | Ref (1.0) | 0.928 (0.865 - 0.995) | 0.721 (0.650 - 0.801) |
| Euploid embryos | Ref (1.0) | 0.854 (0.799 - 0.912) | 0.523 (0.482 - 0.568) |
| Aneuploid proportion (%) | Ref (1.0) | 0.951 (0.904 to 1.000) | 2.132 (2.009 to 2.261) |
| Mosaic proportion (%) | Ref (1.0) | 1.064 (1.002 to 1.130) | 0.735 (0.667 to 0.809) |
| Euploid proportion (%) | Ref (1.0) | 0.980 (0.946 to 1.014) | 0.533 (0.502 to 0.565) |

Poisson regression models were used for count outcomes, and Poisson regression models with offset(log(total)) were used for proportion outcomes, with cluster-robust standard errors by center. All models were adjusted a priori for female age and additionally adjusted for male age. Results are reported as RRs (95% CIs), with PGT-A alone as the reference.

**Supplementary Table 2.** Association between ploidy and outcomes of PGT-A, PGT-M+PGT-A, and PGT-SR with additional adjustment for season

| Ploidy | PGT-A (N=7635) | PGT-M+PGT-A (N=5874) | PGT-SR (N=2447) |
| --- | --- | --- | --- |
| Biopsied blastocysts | Ref (1.0) | 0.866 (0.821 - 0.914) | 0.931 (0.877 - 0.990) |
| Aneuploid embryos | Ref (1.0) | 0.820 (0.760 - 0.885) | 1.979 (1.824 - 2.146) |
| Mosaic embryos | Ref (1.0) | 0.926 (0.863 - 0.992) | 0.684 (0.610 - 0.767) |
| Euploid embryos | Ref (1.0) | 0.850 (0.795 - 0.908) | 0.495 (0.456 - 0.537) |
| Aneuploid proportion (%) | Ref (1.0) | 0.952 (0.905 to 1.001) | 2.149 (2.020 to 2.286) |
| Mosaic proportion (%) | Ref (1.0) | 1.065 (1.003 to 1.131) | 0.731 (0.663 to 0.806) |
| Euploid proportion (%) | Ref (1.0) | 0.979 (0.945 to 1.014) | 0.529 (0.499 to 0.562) |

Poisson regression models were used for count outcomes, and Poisson regression models with offset(log(total)) were used for proportion outcomes, with cluster-robust standard errors by center. All models were adjusted a priori for female age and additionally adjusted for treatment season. Results are reported as RRs (95% CIs), with PGT-A alone as the reference.

**Supplementary Table 3.** Association between ploidy and outcomes of PGT-A, PGT-M+PGT-A, and PGT-SR with adjustment for female age, male age, and season.

| Ploidy | PGT-A (N=7635) | PGT-M+PGT-A (N=5874) | PGT-SR (N=2447) |
| --- | --- | --- | --- |
| Biopsied blastocysts | Ref (1.0) | 0.866 (0.821 - 0.914) | 0.931 (0.876 - 0.989) |
| Aneuploid embryos | Ref (1.0) | 0.820 (0.760 - 0.885) | 1.979 (1.824 - 2.146) |
| Mosaic embryos | Ref (1.0) | 0.925 (0.863 - 0.992) | 0.684 (0.609 - 0.767) |
| Euploid embryos | Ref (1.0) | 0.849 (0.794 - 0.908) | 0.495 (0.456 - 0.537) |
| Aneuploid proportion (%) | Ref (1.0) | 0.952 (0.905 to 1.001) | 2.150 (2.020 to 2.288) |
| Mosaic proportion (%) | Ref (1.0) | 1.065 (1.002 to 1.131) | 0.731 (0.663 to 0.805) |
| Euploid proportion (%) | Ref (1.0) | 0.978 (0.944 to 1.013) | 0.529 (0.499 to 0.562) |

Poisson regression models were used for count outcomes, and Poisson regression models with offset(log(total)) were used for proportion outcomes, with cluster-robust standard errors by center. Models were adjusted for female age, male age, and treatment season. Results are reported as RRs (95% CIs), with PGT-A alone as the reference.

**Supplementary Table 4.** Age-stratified association between PGT strategy and cycle-level ploidy outcomes.

| Ploidy | PGT-A alone | PGT-M+A | PGT-SR |
| --- | --- | --- | --- |
| <35 |  |  |  |
| Non-euploidy | 289 (11.3%) | 624 (14.4%) | 593 (31.1%) |
|  | Ref (1.0) | 1.28 (1.13 - 1.44) | 2.76 (2.38 - 3.19) |
| at least one euploid | 2273 (88.7%) | 3710 (85.6%) | 1312 (68.9%) |
|  | Ref (1.0) | 0.96 (0.95 - 0.98) | 0.78 (0.75 - 0.81) |
| at least one mosaic | 1141 (44.5%) | 1787 (41.2%) | 680 (35.7%) |
|  | Ref (1.0) | 0.93 (0.87 - 0.99) | 0.80 (0.74 - 0.86) |
| 35-40 |  |  |  |
| Non-euploidy | 978 (27.4%) | 365 (26.7%) | 217 (44.1%) |
|  | Ref (1.0) | 0.97 (0.86 - 1.10) | 1.61 (1.41 - 1.84) |
| at least one euploid | 2590 (72.6%) | 1001 (73.3%) | 275 (55.9%) |
|  | Ref (1.0) | 1.01 (0.96 - 1.06) | 0.77 (0.71 - 0.84) |
| at least one mosaic | 1119 (31.4%) | 449 (32.9%) | 127 (25.8%) |
|  | Ref (1.0) | 1.05 (0.96 - 1.15) | 0.82 (0.71 - 0.95) |
| >40 |  |  |  |
| Non-euploidy | 876 (58.2%) | 111 (63.8%) | 39 (78%) |
|  | Ref (1.0) | 1.10 (0.95 - 1.26) | 1.34 (1.15 - 1.56) |
| at least one euploid | 629 (41.8%) | 63 (36.2%) | 11 (22%) |
|  | Ref (1.0) | 0.87 (0.69 - 1.09) | 0.53 (0.32 - 0.87) |
| at least one mosaic | 285 (18.9%) | 27 (15.5%) | 7 (14%) |
|  | Ref (1.0) | 0.82 (0.54 - 1.24) | 0.74 (0.36 - 1.51) |

Relative risks and 95% CIs were estimated using Poisson regression models with robust standard errors clustered by center, with PGT-A alone as the reference group. Analyses were performed separately within each female age stratum (<35, 35–40, and >40 years).

Binary outcomes were defined at the cycle level. “Non-euploidy” was defined as the absence of euploid embryos per cycle. “At least one euploid” and “at least one mosaic” were defined as the presence versus absence of ≥1 euploid embryo or ≥1 mosaic embryo per cycle, respectively.

**Supplementary Table 5.** Association between PGT strategy and age-stratified count outcomes of blastocyst biopsy and embryo ploidy.

| Ploidy | PGT-A alone | PGT-M+A | PGT-SR |
| --- | --- | --- | --- |
| <35 |  |  |  |
| Biopsied blastocysts | 4.26 (4.16 - 4.37) | 3.83 (3.75 - 3.92) | 4.40 (4.26 - 4.53) |
|  | Ref (1.0) | 0.90 (0.85 to 0.95) | 1.03 (0.97 to 1.10) |
| Aneuploid embryos | 0.98 (0.94 - 1.03) | 0.87 (0.84 - 0.91) | 2.43 (2.34 - 2.53) |
|  | Ref (1.0) | 0.89 (0.81 to 0.98) | 2.48 (2.23 to 2.75) |
| Mosaic embryos | 0.62 (0.59 - 0.66) | 0.60 (0.57 - 0.62) | 0.48 (0.44 - 0.51) |
|  | Ref (1.0) | 0.95 (0.87 to 1.04) | 0.77 (0.69 to 0.85) |
| Euploid embryos | 2.66 (2.58 - 2.74) | 2.37 (2.30 - 2.43) | 1.49 (1.42 - 1.56) |
|  | Ref (1.0) | 0.89 (0.84 to 0.95) | 0.56 (0.52 to 0.60) |
| 35-37 |  |  |  |
| Biopsied blastocysts | 3.67 (3.55 - 3.79) | 3.20 (3.05 - 3.35) | 3.55 (3.28 - 3.83) |
|  | Ref (1.0) | 0.87 (0.80 to 0.95) | 0.97 (0.89 to 1.05) |
| Aneuploid embryos | 1.23 (1.17 - 1.29) | 1.09 (1.01 - 1.17) | 2.17 (1.96 - 2.37) |
|  | Ref (1.0) | 0.89 (0.79 to 1.00) | 1.76 (1.58 to 1.95) |
| Mosaic embryos | 0.46 (0.43 - 0.50) | 0.45 (0.40 - 0.49) | 0.34 (0.28 - 0.40) |
|  | Ref (1.0) | 0.96 (0.86 to 1.08) | 0.73 (0.61 to 0.88) |
| Euploid embryos | 1.98 (1.89 - 2.06) | 1.66 (1.56 - 1.76) | 1.05 (0.91 - 1.19) |
|  | Ref (1.0) | 0.84 (0.77 to 0.92) | 0.53 (0.46 to 0.62) |
| 38-40 |  |  |  |
| Biopsied blastocysts | 3.09 (3.00 - 3.19) | 2.62 (2.44 - 2.79) | 2.85 (2.54 - 3.15) |
|  | Ref (1.0) | 0.85 (0.76 to 0.94) | 0.92 (0.84 to 1.01) |
| Aneuploid embryos | 1.52 (1.46 - 1.58) | 1.25 (1.13 - 1.36) | 1.91 (1.67 - 2.15) |
|  | Ref (1.0) | 0.82 (0.71 to 0.94) | 1.25 (1.12 to 1.41) |
| Mosaic embryos | 0.33 (0.30 - 0.35) | 0.33 (0.27 - 0.38) | 0.25 (0.16 - 0.34) |
|  | Ref (1.0) | 1.00 (0.84 to 1.19) | 0.77 (0.54 to 1.12) |
| Euploid embryos | 1.24 (1.18 - 1.31) | 1.04 (0.93 - 1.16) | 0.69 (0.56 - 0.81) |
|  | Ref (1.0) | 0.84 (0.72 to 0.97) | 0.55 (0.47 to 0.65) |
| 41-42 |  |  |  |
| Biopsied blastocysts | 2.71 (2.58 - 2.83) | 2.30 (2.04 - 2.56) | 2.00 (1.61 - 2.39) |
|  | Ref (1.0) | 0.85 (0.74 to 0.98) | 0.74 (0.62 to 0.88) |
| Aneuploid embryos | 1.67 (1.58 - 1.75) | 1.41 (1.20 - 1.61) | 1.52 (1.23 - 1.80) |
|  | Ref (1.0) | 0.84 (0.72 to 0.99) | 0.91 (0.78 to 1.06) |
| Mosaic embryos | 0.24 (0.21 - 0.28) | 0.20 (0.12 - 0.28) | 0.14 (0.01 - 0.27) |
|  | Ref (1.0) | 0.84 (0.57 to 1.23) | 0.57 (0.24 to 1.36) |
| Euploid embryos | 0.80 (0.72 - 0.87) | 0.69 (0.52 - 0.86) | 0.34 (0.12 - 0.57) |
|  | Ref (1.0) | 0.87 (0.65 to 1.16) | 0.43 (0.24 to 0.78) |
| >42 |  |  |  |
| Biopsied blastocysts | 2.33 (2.19 - 2.47) | 1.85 (1.50 - 2.21) | 2.00 (0.97 - 3.03) |
|  | Ref (1.0) | 0.79 (0.66 to 0.96) | 0.86 (0.51 to 1.45) |
| Aneuploid embryos | 1.58 (1.48 - 1.68) | 1.61 (1.30 - 1.92) | 1.67 (0.61 - 2.73) |
|  | Ref (1.0) | 1.02 (0.82 to 1.26) | 1.05 (0.56 to 1.98) |
| Mosaic embryos | 0.18 (0.14 - 0.22) | 0.07 (0.0 - 0.15) | 0.14 (0.0 - 0.30) |
|  | Ref (1.0) | 0.41 (0.14 to 1.19) | 0.79 (0.27 to 2.36) |
| Euploid embryos | 0.57 (0.49 - 0.66) | 0.17 (0.02 - 0.32) | 0.19 (0.0 - 0.41) |
|  | Ref (1.0) | 0.30 (0.11 to 0.81) | 0.33 (0.10 to 1.06) |

Descriptive data are presented as mean (95% confidence interval) for the number of biopsied blastocysts and numbers of euploid, aneuploid, and mosaic embryos per cycle. Poisson regression models were used to compare count outcomes across PGT strategies within each female age subgroup, with cluster-robust standard errors by center. Results are reported as rate ratios with 95% CIs, using PGT-A alone as the reference.

**Supplementary Table 6.** Probability of achieving at least one euploid embryo per cycle among PGT-A alone and PGT-SR subtypes by female age and embryos for biopsy.

|  | PGT-A alone %(SE) | PGT-SR %(SE) | | | p-value comparing to PGT-alone | | |
| --- | --- | --- | --- | --- | --- | --- | --- |
| Age | n = 7635 | Balanced SR=1840 | Cx/Unbal-SR=27 | Sex-Aneu=199 | Balanced SR | Cx/Unbal-SR | Sex-Aneu |
| <35 | N = 2562 | 1426 | 21 | 158 |  |  |  |
| Combined | 88.72 (0.62) | 65.78 (1.26) | 42.86 (10.8) | 78.48 (3.27) | <0.001 | 0.002 | 0.005 |
| 1-3 EMFB | 77.16 (1.23) | 45.3 (1.94) | 30.77 (12.8) | 60.81 (5.67) | <0.001 | 0.012 | 0.007 |
| 4-6 EMFB | 97.46 (0.52) | 77.21 (1.97) | 1/3 | 93.55 (3.12) | <0.001 | - | 0.223 |
| >7 EMFB | 99.8 (0.2) | 92.36 (1.5) | 80 (17.89) | 95.45 (4.44) | <0.001 | 0.204 | 0.355 |
| 35-40 | N = 3568 | 378 | 6 | 38 |  |  |  |
| Combined | 72.59 (0.75) | 53.97 (2.56) | 33.33 (19.25) | 73.68 (7.14) | <0.001 | 0.214 | 0.878 |
| 1-3 EMFB | 60.25 (1.04) | 40 (3.13) | 0/4 | 60 (10.95) | <0.001 | - | 0.979 |
| 4-6 EMFB | 89.79 (0.96) | 76.53 (4.28) | 2/2 | 87.5 (8.27) | 0.011 | - | 0.775 |
| >7 EMFB | 99.73 (0.27) | 88.57 (5.38) | - | 2/2 | 0.053 | - | - |
| >40 | N = 1505 | 36 | 0 | 3 |  |  |  |
| Combined | 41.79 (1.27) | 13.89 (5.76) | - | 0/3 | 0.012 | - | - |
| 1-3 EMFB | 31.44 (1.36) | 11.76 (5.53) | - | 0/3 | 0.042 | - | - |
| 4-6 EMFB | 72.79 (2.65) | 1/1 | - | - | - | - | - |
| >7 EMFB | 95.08 (2.77) | 0/1 | - | - | - | - | - |

Balanced SR = balanced structural rearrangements; Cx/Unbal-SR = complex or unbalanced structural rearrangements; Sex-Aneu = sex chromosome aneuploidies; EMFB = embryos for biopsy; SE = standard error. Values are presented as the percentage of cycles with at least one euploid embryo, with SEs shown in parentheses. Results are stratified by female age group (<35, 35–40, and >40 years) and number of embryos biopsied per cycle (1–3, 4–6, and >7). Comparisons between each PGT-SR subtype and PGT-A alone were performed within each age-by-EMFB stratum. Relative risks were estimated using Poisson regression models with robust standard errors clustered by center. P values shown are unadjusted. For the three subtype comparisons within each age-by-EMFB stratum, a Bonferroni-adjusted significance threshold of 0.0167 (0.05/3) was used to determine statistical significance (two-sided). A total of 381 PGT-SR cycles with missing or unclassified subtype were excluded from the subtype-stratified analyses and are not shown in the Balanced SR, Cx/Unbal-SR, or Sex-Aneu columns. For strata with zero events or fewer than 5 cycles (N < 5), statistical estimation is unreliable. Consequently, data in these cells are presented as raw counts (n/N) without percentages or standard errors, and corresponding p-values are omitted and marked with dashes.

**Supplementary Figure 1.** Forest plot of unadjusted and age-adjusted relative risks (RRs) for embryo biopsy and ploidy outcomes.

The left and right panels compare the combined PGT-M+PGT-A cohort and the PGT-SR cohort, respectively, against the PGT-A alone reference group. Blue squares represent unadjusted (crude) RRs, while red squares represent age-adjusted RRs, with horizontal lines indicating 95% confidence intervals (CIs). The dashed vertical line indicates an RR of 1.0 (no difference). Count outcomes and proportional outcomes (%) were estimated using Poisson regression models and Poisson models with a log (total embryos) offset, respectively.
